# Human serum albumin as the carrier to fabricate STING-activating peptide nanovaccine for antitumor immunotherapy

**DOI:** 10.1016/j.mtbio.2024.100955

**Published:** 2024-01-14

**Authors:** Aixian Zheng, Zhaoyu Ning, Xiaorong Wang, Zhenli Li, Yupeng Sun, Ming Wu, Da Zhang, Xiaolong Liu, Jianwu Chen, Yongyi Zeng

**Affiliations:** a The United Innovation of Mengchao Hepatobiliary Technology Key Laboratory of Fujian Province, Mengchao Hepatobiliary Hospital of Fujian Medical University, Fuzhou, 350025, PR China; b College of Biological Science and Engineering, Fuzhou University, Fuzhou, 350116, PR China; c Department of Radiotherapy, Fujian Medical University Union Hospital, Fuzhou, 350004, PR China

**Keywords:** Immunotherapy, Peptide nanovaccine, Human serum albumin, Antigenic peptides, MnO_2_

## Abstract

Tumor vaccines are emerging as one of the most promising therapeutic strategies for cancer treatment. With the advantages of low toxicity, convenient production and stable quality control, peptide vaccines have been widely used in preclinical and clinical trials involving various malignancies. However, when used alone, they still suffer from significant challenges including poor stability and immunogenicity as well as the low delivery efficiency, leading to limited therapeutic success. Herein, the STING-activating peptide nanovaccine based on human serum albumin (HSA) and biodegradable MnO_2_ was constructed, which can improve the stability and immunogenicity of antigenic peptides as well as facilitate their uptake by dendritic cells (DCs). Meanwhile, Mn^2+^ degraded from the nanovaccine can activate the STING pathway and further promote DCs maturation. In this way, the prepared nanovaccine can efficiently mediate T-cell immune responses, thereby exerting the effects of tumor prevention and therapy. Moreover, the prepared nanovaccine possesses the advantages of low cost, convenient preparation and good biocompatibility, showing great potential for practical applications.

## Graphical abstract

**Figure:**
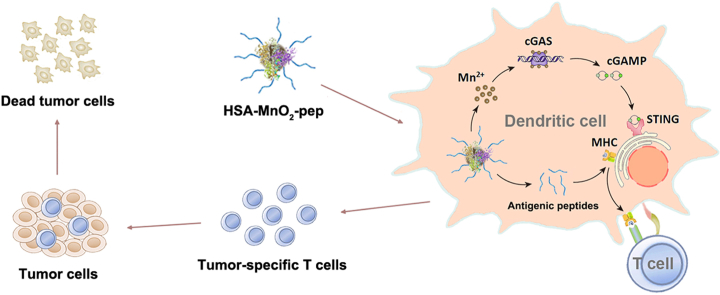

## 1 Introduction

Tumor immunotherapy has been considered as a promising therapeutic strategy by reactivating the immune system to defend and eliminate cancer cells. In the past decades, great efforts have been devoted to the development of effective therapeutic strategies [[1], [2], [3], [4]]. Among them, tumor vaccines have received growing attention with the aim of triggering tumor-specific immunity not only allows for establishing immunological memory to prevent cancer but also for eliciting antitumor immune responses to directly eliminate cancer cells [[5], [6], [7]]. Antigenic peptide based vaccines are one of the most promising kinds of tumor vaccines, which have been widely used in preclinical and clinical trials involving various malignancies. Compared with nucleic acid vaccines and full-length protein vaccines, peptide vaccines possess the advantages of low toxicity, convenient production and stable quality control. However, when used alone, they still suffer from critical challenges, such as poor stability and immunogenicity as well as the low delivery efficiency, making them cannot mediate effective antitumor immunity [[8], [9], [10], [11]].

Up to now, a variety of nanomaterials ranging from inorganic nanoparticles to organic polymers have been applied as delivery carriers to construct tumor nanovaccines [[12], [13], [14], [15]]. In which, antigenic peptides can be delivered by nanomaterials into antigen-presenting cells (APCs), especially dendritic cells (DCs), which were subsequently processed and presented via the major histocompatibility complex (MHC) to elicit robust antitumor T-cell immunity. Despite the great potential, the clinical trials of many nanovaccines have encountered serious obstacles due to limited antigen loading capacity and potential long-term toxicity. Thus, there is still a need for an efficient and biocompatible carrier for peptide based tumor vaccines. Human serum albumin (HSA) is the most abundant plasma protein with high water solubility. As an important biological reagent, HSA exhibits excellent stability and native biocompatibility, which has been widely used in experimental research and clinical practice [[16], [17], [18], [19]]. It is well known that proteins such as bovine serum albumin (BSA) and ovalbumin (OVA) can be used as carrier proteins to improve the immunogenicity of the antigens with low molecular weight, which can be applied in antibody preparation or other immune studies [20,21]. Thus, we would like to use HSA as the delivery carrier of antigenic peptides, hoping to overcome the aforementioned issues of poor stability and weak immunogenicity.

On the other hand, adjuvants play an important role in tumor vaccines for eliciting sufficient immune responses, and thus greatly enhance the therapeutic effects of peptide vaccines [[22], [23], [24], [25]]. It has been reported that Mn^2+^ can promote the activation of intracellular stimulator of interferon genes (STING) pathway, which can further promote DC maturation and antigen presentation, thereby enhancing antitumor T-cell immune responses [[26], [27], [28], [29], [30]]. Thus, the combination of Mn^2+^ and peptide vaccine is expected to achieve highly efficient antitumor effects. Interestingly, HSA can serve as both template and reducing agent to prepare MnO_2_ through the redox reaction between HSA and KMnO_4_ [31,32]. Moreover, MnO_2_ can be completely degraded into Mn^2+^ in cells [33,34], which can not only avoid long-term toxicity, but also play its role as immunologic adjuvant.

Herein, HSA was used as the carrier to prepare MnO_2_, which can be further linked with tumor antigenic peptides to prepare STING-activating peptide nanovaccine (HSA-MnO_2_-pep) for tumor prevention and therapy. The conjugation between HSA-MnO_2_ and tumor antigenic peptides can be achieved through the assistance of succinimidyl-4-(N-maleimidomethyl)cyclohexane-1-carboxylate (SMCC), which is a bifunctional coupling agent containing N-hydroxysuccinimide ester and maleimide [[35], [36], [37]]. The presence of HSA-MnO_2_ can improve the stability and immunogenicity of antigenic peptides as well as facilitate their uptake by APCs. Meanwhile, MnO_2_ contained in the nanovaccine can be degraded into Mn^2+^, which can activate the STING pathway and further promote DCs maturation, thereby effectively inducing antigen-specific T-cell immune responses to defend and eliminate tumor cells. This work is expected to provide a simple and high efficient peptide nanovaccine for tumor immunotherapy.

## 2 Materials and methods

### 2.1 Reagents and apparatus

Ethylenediamine tetraacetic acid (EDTA), 4 % paraformaldehyde, HSA, Coomassie brilliant blue G250 and hematoxylin and eosin (H&E) staining kit were purchased from Beijing Solarbio Technology Co., Ltd (China). 4′,6-Diamidino-2-phenylindole Dihydrochloride (DAPI), trypan blue and glutathione (GSH) were obtained from Sigma-Aldrich Chemical Co. (USA). SMCC and 5,5′-dithiobis (2-nitrobenzoic acid) (DTNB) were purchased from Adamas-beta (Shanghai, China). BCA protein quantitation kit and cell freezing medium were purchased from Beyotime Biotechnology Co., Ltd. (China). DMEM medium, RPMI 1640 medium, phosphate buffer saline (PBS), fetal bovine serum (FBS), penicillin and streptomycin were purchased from Gibco (USA). Interleukin-4 (IL-4) and Granulocyte-macrophage colony-stimulating factor (GM-CSF) were purchased from Wolcavi Biotech (Beijing, China). Mouse interferon-gamma (IFN-γ) enzyme-linked immunospot (ELISpot) plus kit was purchased from Mabtech (Sweden). Anti-CD11c-APC, anti-CD80-PE and anti-CD86-PE-Cy7 were purchased from Thermo Fisher Scientific (USA). Anti-CD3-APC and anti-CD8-PE were purchased from BioLegend, Inc (USA). Mouse IFN-γ enzyme-linked immunosorbent assay (ELISA) kit, mouse interferon beta (IFN-β) ELISA kit and mouse tumor necrosis factor alpha (TNF-α) ELISA kit were purchased from Fankewei (Shanghai, China).

### 2.2 Cell culture and animals

The mouse liver cancer cells (Hepa1-6) and DC2.4 cells were cultured in DMEM medium at 37 °C in a humidified atmosphere (5 % CO_2_), which was supplemented with 10 % fetal bovine serum and 1 % streptomycin/penicillin. Male C57BL/6 mice were purchased from China Wushi, Inc (Shanghai, China). All animal experiments have been approved by Animal Ethics Committee of Mengchao Hepatobiliary Hospital of Fujian Medical University (the approval number is MCHH-AEC-2022-02). Meanwhile, all animal procedures were conducted in accordance with the Guidelines for Care and Use of Laboratory Animals.

### 2.3 The identification of tumor antigenic peptide and immunogenicity validation

Firstly, 361 liver cancer tissue samples and 50 normal liver tissue samples were extracted from the Cancer Genome Atlas (TCGA) database and used for the screen of tumor-associated antigen. In addition, we collected the cancerous tissues and their paired paracancerous tissues of 72 liver cancer patients, and analyzed the expression level of Mitogen-Activated Protein Kinase 3 (MAPK3) gene through transcriptome sequencing. All collection and usage of human samples were in accordance with the principles of the Declaration of Helsinki and approved by the Institution Review Board of Mengchao Hepatobiliary Hospital of Fujian Medical University (the approval number is 2018_003_01). Written consents were received from all participating patients. NetMHC-4.0 was used to predict the binding affinity between peptide and H2-Kb allele and thus used for immunogenicity evaluation of MAPK3 derived peptides. In addition, most MHC I molecules have a strong preference for binding peptides with 9 amino acids. Thus, MAPK3 derived peptides were designed based on every 9 amino acids.

### 2.4 The preparation and characterization of HSA-pep

In order to conjugate tumor antigenic peptide to HSA, cysteine was added at the N-terminal of the antigenic peptide, which was covalently linked to HSA through SMCC. Briefly, 10 mg of HSA was dissolved in 1 mL of phosphate buffered saline (PBS), and 5 mg of SMCC was dissolved in 1 mL of N,N-dimethylformamide (DMF). Then, 1 mL of HSA solution was reacted with 300 μL of SMCC at room temperature for 2 h. In order to remove DMF and unreacted SMCC, HSA-SMCC was obtained by ultrafiltration at 7000 g using a 10 KD Millipore and re-dispersed in 1 mL PBS. Subsequently, 500 μL of HSA-SMCC was mixed with antigen peptides at a molar ratio of 1:30. After reaction at room temperature for 4 h, HSA-pep was obtained by ultrafiltration at 7000 g using a 10 KD Millipore and re-dispersed in 1 mL PBS.

To confirm the successful preparation of HSA-pep, HSA and HSA-pep were determined by 10 % sodium dodecyl sulfate-polyacrylamide gel electrophoresis (SDS-PAGE) and Coomassie blue staining. After destaining, the image was visualized by a Gel Doc XR imaging system (Bio-Rad). DTNB contained Ellman's reagent was used to quantify free thiol groups in the peptide solution, which was prepared by dissolving 4 mg of DTNB in 1 mL of 0.1 M phosphate buffer (pH = 8) containing 1 mM ethylenediaminetetraacetic acid (EDTA). For the analysis of free thiols, 20 μL of different samples were added with 4 μL of Ellman's reagent and 176 μL of Ellman's buffer. After reacting at room temperature for 15 min, the absorption spectra in the range of 250 nm–650 nm were measured. Meanwhile, the linear relationship between peptide concentrations and the absorbance intensities at 412 nm was obtained for calculating the number of peptides conjugated to each HSA.

### 2.5 The preparation and characterization of HSA-MnO_2_-pep

The synthesis of HSA-MnO_2_ was firstly optimized. Briefly, different concentrations of KMnO_4_ were mixed with 4 mg mL^−1^ HSA and then reacted at 37 °C for 2 h. By measuring the hydrodynamic sizes and absorption spectra, the final concentration of KMnO_4_ was selected as 6 mM. After reaction, HSA-MnO_2_ was obtained by ultrafiltration at 7000 g using a 10 KD Millipore and then re-dispersed in PBS. Then, HSA-MnO_2_ was mixed with SMCC at a molar ratio of 1:30. After reaction at room temperature for 2 h, HSA-MnO_2_-SMCC was obtained by ultrafiltration at 7000 g using a 10 KD Millipore and re-dispersed in PBS, which was further mixed with antigenic peptides at a molar ratio of 1:30. After reaction at room temperature for 4 h, HSA-MnO_2_-pep was obtained by ultrafiltration at 7000 g using a 10 KD millipore and re-dispersed in PBS, followed by storing at 4 °C for future use. The successful preparation of HSA-MnO_2_-pep was characterized by atomic force microscope (AFM) imaging, X-ray photoelectron spectroscopy (XPS), dynamic light scattering (DLS) analysis as well as Ellman's reagent based analysis.

### 2.6 GSH-responsive degradation of HSA-MnO_2_-pep

GSH can induce the degradation of HSA-MnO_2_-pep into Mn^2+^, and this process can be characterized by the change of absorption spectra and the generation of Mn^2+^ measured by inductively coupled plasma atomic emission spectrometer (ICP-OES). Specifically, 200 μL of 5 mg mL^−1^ HSA-MnO_2_-pep was reacted with 10 μL of 0.4 M GSH, and the solution of HSA-MnO_2_-pep added with 10 μL of PBS was used as the control. Then, the absorption spectra of the solution with or without GSH treatment were measured in the range of 200–800 nm. To confirm the degradation of MnO_2_ into Mn^2+^, the solution with or without GSH treatment were subjected to ultrafiltration using a 10 KD millipore, and the concentrations of Mn^2+^ in the solution of lower layers were detected by ICP-OES.

### 2.7 The uptake of the nanovaccine by DC2.4 cells

For imaging purposes, HSA-pep-FAM and HSA-MnO_2_-pep-FAM were prepared similar to the preparation of HSA-pep and HSA-MnO_2_-pep. The fluorescent spectra and fluorescence imaging under 488 nm laser excitation were conducted to verify the successful conjugation of peptide-FAM to HSA or HSA-MnO_2_. Meanwhile, the linear relationship between peptide-FAM concentrations and the fluorescent intensities at 520 nm was obtained for calculating the number of peptide-FAM conjugated to each HSA or HSA-MnO_2_.

Next, C57BL/6-derived dendritic cells (DC2.4 cells) or murine bone marrow-derived dendritic cells (BMDCs) were seeded in 15 mm confocal dishes at a density of 2.5 × 10^5^ cells per well, which were cultured in DMEM medium containing 10 % FBS. The cells were then incubated with 1 mL of peptide-FAM, HSA-pep-FAM and HSA-MnO_2_-pep-FAM respectively at 37 °C for 4 h with same concentrations of peptide-FAM added to each dish. Subsequently, the cells were fixed with 4 % paraformaldehyde for 15 min and then stained with DAPI for 15 min. After washed with PBS, the images of the cells were recorded by a confocal fluorescence microscope.

We further used flow cytometry to analyze the uptake of nanovaccines by DC2.4 cells or BMDCs. Briefly, DC2.4 cells were seeded in 24 well plates at a density of 5 × 10^5^ cells per well, which were cultured in DMEM medium containing 10 % FBS. The cells were then incubated with peptide-FAM, HSA-pep-FAM or HSA-MnO_2_-pep-FAM respectively at 37 °C for 4 h. After that, the cells were obtained by centrifugation at 800*g* for 5 min and then dispersed in PBS, follow by analyzing the fluorescence signals through flow cytometry.

### 2.8 HSA-MnO_2_-pep stimulates the maturation of BMDCs

BMDCs were extracted from tibias and femurs of C57BL/6 mouse according to the previously reported method [25]. Briefly, both ends of the bones were cut off with scissors and the cells in the bones were blown out by a sterile syringe. The cells were collected by passing through 40 μm filter and centrifuging at 800 g for 5 min, followed by incubating in red blood cell lysis buffer for 10 min. After centrifugation to remove the lysed red blood cells, the obtained cells were cultured in RPMI 1640 medium containing 20 ng mL^−1^ GM-CSF, 10 ng mL^−1^ IL-4, 10 % FBS and 1 % penicillin-streptomycin for 5 days. After that, non-adherent cells, also known as immature BMDCs, were harvested for subsequent experiments.

Next, immature BMDCs were seeded in a 24-well plate at a density of 5 × 10^5^ cells per well, and then incubated with 1 mL of PBS, LPS, HSA, peptide, HSA-MnO_2_, HSA-pep and HSA-MnO_2_-pep respectively at 37 °C for 48 h. The concentration of peptides was 30 μM. Subsequently, BMDCs were harvested and blocked with 5 % BSA for 15 min, and then collected by centrifugation at 800*g* for 5 min. After that, the collected cells were stained with 0.5 % BSA diluted anti-CD11c-APC, anti-CD80-PE and anti-CD86-PE-Cy7 for 30 min. After washing with PBS, the proportion of mature BMDCs was analyzed by flow cytometry.

Meanwhile, the BMDCs after different treatments were also collected for western blot analysis of tank-binding kinase 1 (TBK1) and phosphorylated TBK1 (p-TBK1). Briefly, the cells in each group were lysed in radioimmunoprecipitation assay (RIPA) buffer containing halt protease inhibitor cocktail. After centrifuging at 12000 g for 10 min, the supernatant was collected and quantified by the bicinchoninic acid (BCA) assay. The obtained samples were separated by 10 % sodium dodecyl-sulfate polyacrylamide gel electrophoresis (SDS-PAGE). After transferred to the nitrocellulose (NC) membrane, the expressions of TBK1 and p-TBK1 were analyzed by immunoblotting using the corresponding primary and secondary antibodies. After ultrasound fragmentation of the BMDCs after different treatments for 24 h, the supernatant was also collected by centrifugation and then used for the analysis of IFN-β by corresponding ELISA kit.

### 2.9 HSA-MnO_2_-pep stimulates the maturation of DCs in mouse lymph nodes

The accumulation of nanovaccines in mouse lymph nodes was recorded by ex vivo fluorescence imaging. C57BL/6 mice were subcutaneously injected with PBS, peptide-FAM and HSA-MnO_2_-pep-FAM respectively in their groin. 18 h later, ex vivo fluorescence images of the lymph nodes isolated from the mice were recorded. The excitation wavelength was at 488 nm.

To verify that the nanovaccine can stimulate the maturation of DCs in lymph nodes, C57BL/6 mice were subcutaneously injected with 100 μL of PBS, peptides, HSA-MnO_2_, HSA-pep and HSA-MnO_2_-pep respectively in their groin at a dose of 100 μg peptide per injection. And the mice were injected every three days for a total of three injections. Three days after last injection, the lymph nodes of three mice in each group were collected and grinded to prepare cell suspensions. After passing through 40 μm filter and centrifuging at 800 g for 5 min, the cells were harvested and blocked with 5 % BSA for 15 min. After that, the collected cells were stained with 0.5 % BSA diluted anti-CD11c-APC, anti-CD80-PE and anti-CD86-PE-Cy7 for 30 min, followed by PBS washing and flow cytometry analysis.

### 2.10 ELISPOT to verify the antigen-specific reactivity

IFN-γ secretion from mouse splenic T cells was detected by enzyme linked immunospot assay (ELISPOT) to verify the antigen-specific reactivity. Briefly, C57BL/6 mice were subcutaneously injected with 100 μL of PBS, peptides and HSA-MnO_2_-pep respectively in their groin at a dose of 100 μg peptide per injection. The mice were injected every three days for a total of three injections. Three days after last injection, the spleens of three mice in each group were collected, and the cells were blown out by a sterile syringe. The splenic monocytes were collected by passing through 40 μm filter and using Ficoll-based density gradient centrifugation at 800*g* for 20 min. On the other hand, immature BMDCs were obtained and seeded in the ELISPOT plate at a density of 1 × 10^4^ cells per well. After activated with 4 μg of antigenic peptides per well for 36 h, the BMDCs were further incubated with 1 × 10^5^ of the collected splenic monocytes respectively for 36 h. Then, the secreted IFN-γ was labelled with biotinylated mAb R4-6A2 as well as streptavidin-ALP. After color reaction, the formed IFN-γ spots were imaged and analyzed by Elispot Reader.

### 2.11 Tumor prevention by HSA-MnO_2_-pep

To investigate the tumor prevention effect of HSA-MnO_2_-pep, healthy C57BL/6 mice were subcutaneously injected with 100 μL of PBS, peptides, HSA-MnO_2_, HSA-pep and HSA-MnO_2_-pep respectively in their groin at a dose of 100 μg peptide per injection. The mice were injected every three days for a total of three injections. Four days after last injection, 3 × 10^6^ Hepa1-6 cells were subcutaneously injected into the back of each mouse. Then, the body weight and tumor volume of each mouse were measured every two days. The tumor volume was calculated to be LW^2^/2, where L and W were the length and width of tumor respectively. The mice were euthanized when the tumor volume of which up to 1200 mm^3^.

### 2.12 In vivo antitumor effect of HSA-MnO_2_-pep

To investigate the antitumor effect of HSA-MnO_2_-pep, tumor-bearing mice were firstly obtained by subcutaneously injection of 3 × 10^6^ Hepa1-6 cells into the back of each mouse. Seven days later, the mice were randomly divided into five groups, which were subcutaneously injected with 100 μL of PBS, peptides, HSA-MnO_2_, HSA-pep and HSA-MnO_2_-pep respectively in their groin at a dose of 100 μg peptide per injection. The mice were injected every three days for a total of three injections. Then, the body weight and tumor volume of each mouse were measured every two days. The mice were euthanized when the tumor volume of which up to 1200 mm^3^.

To further confirm the antitumor effect, three mice in each group were euthanized and the tumor of each mouse was obtained on the third day after last injection. Subsequently, a portion of each tumor was weighed and digested by hyaluronidase, collagenase IV and DNase I at 37 °C for 2 h. The single cell suspensions were collected by passing through 40 μm filter and using Ficoll-based density gradient centrifugation at 800*g* for 20 min. The cells were blocked with 5 % BSA for 15 min, and then collected by centrifugation at 800*g* for 5 min. After that, the collected cells were stained with 0.5 % BSA diluted anti-CD3-APC and anti-CD8-PE for 30 min. After washing with PBS, the infiltration of CD8+T cells in the tumor was evaluated by flow cytometry. Furthermore, a portion of each tumor was weighed and homogenized with 1 mL PBS through a fully automatic sample rapid grinder. The supernatant of the samples were obtained by centrifugation at 800*g*, and the concentrations of IFN-γ and TNF-α in which were analyzed by corresponding ELISA kits. Moreover, the tumors from five groups were also obtained on the third day after last injection, which were further fixed in formalin solution as well as embedded in paraffin. The tumor sections were then deparaffinized and subjected to hematoxylin-eosin (H&E) and Ki67 staining.

### 2.13 Biosafety assessment

To assess the biosafety of the prepared nanovaccine, the major organs and fresh blood specimens of three mice in each group were collected on the third day after last injection. The heart, liver, spleen, lung and kidney from each group were fixed in formalin solution and embedded in paraffin. The tumor sections were obtained and then deparaffinized for H&E staining. Sera were collected from the blood specimens by centrifugation at 7000 rpm, which were used for the analysis of serum biochemical indicators through an automatic biochemical analyzer (CX5, Beckman, USA). The biochemical indicators contained aspartate aminotransferase (AST), alanine aminotransferase (ALT), alkaline phosphatase (ALP), glucose (GLU), creatinine (CREA) and urea nitrogen (UREA).

### 2.14 Statistical analysis

The data in this manuscript were presented in the form of mean ± standard deviation. The statistical differences were analyzed using one-way analysis of variance (ANOVA), which were divided into *P < 0.05, **P < 0.01 and ***P < 0.001. *P < 0.05 was considered as significant.

## 3 Results and discussion

### 3.1 Screening of tumor antigenic peptide

The TCGA database is an authoritative cancer gene database, which collates up to 33 kinds of cancer related biological data, including genome variation, transcriptome expression and methylation. TCGA was used to screen potential tumor-associated antigens that are highly expressed in tumor tissue and lowly expressed in normal tissue. 361 liver cancer tissue samples and 50 normal liver tissue samples were firstly screened from TCGA database. After analysis, it can be found that the expression levels of MAPK3 gene in liver cancer tissue were significantly higher than that in normal liver tissue (Fig. S1a). On the other hand, cancerous tissues and their paired adjacent tissues of 72 liver cancer patients were collected for analyzing the expression levels of MAPK3 gene by transcriptome sequencing. As shown in Fig. S1b, the expression level of MAPK3 gene in cancerous tissues of liver cancer patients was obviously higher than that in their paired adjacent tissues, indicating that MAPK3 may be a potential tumor-associated antigen. NetMHC-4.0 program can be used to predict the binding affinity between peptides and MHC class I molecules, thus evaluating the immunogenicity of MAPK3 derived peptides. As shown in Table S1, the peptide located at 277–285 in mouse MAPK3 exhibited the highest affinity with H2-Kb allele. As a proof-of-concept, the peptide (MKARNYLQSLPSKTKVA) containing this sequence was used as the tumor antigenic peptide to construct peptide nanovaccine in subsequent experiments.

### 3.2 The preparation and characterization of HSA-pep and HSA-MnO_2_-pep

**Fig. 1:**
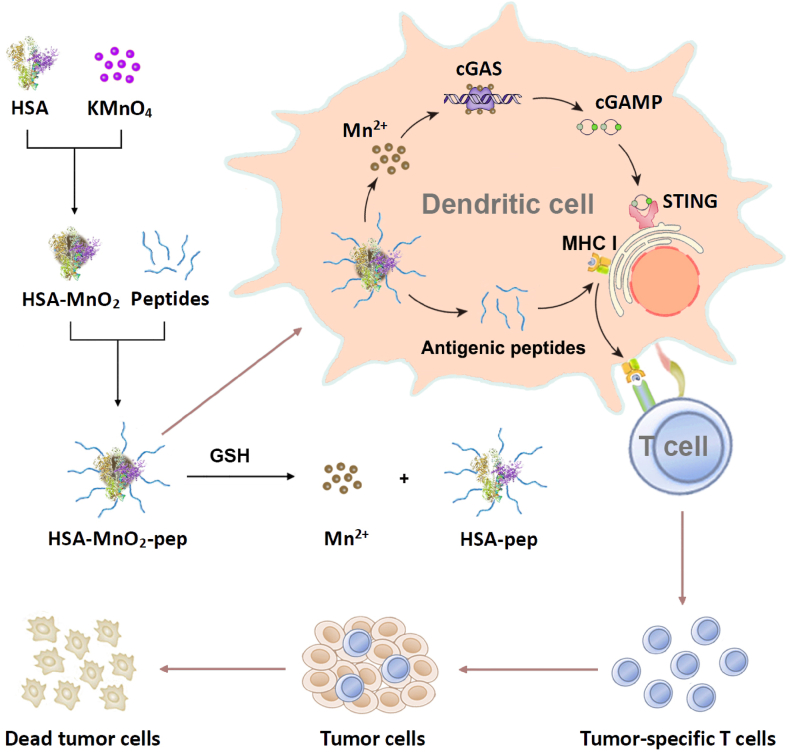
Schematic illustration of the preparation of STING-activating peptide nanovaccine and its application in antitumor immunotherapy.

**Fig. 2:**
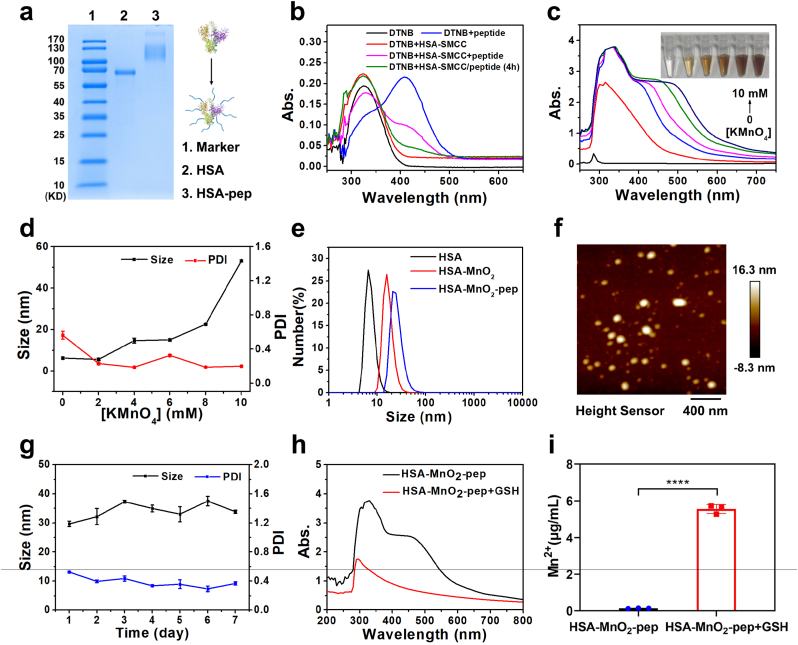
(a) Coomassie blue staining of HSA and HSA-pep; (b) absorption spectra of DTNB after reaction with HSA-SMCC or peptides under different conditions; (c) absorption spectra and color changes of HSA after reaction with different concentrations of KMnO_4_ for 2 h; (d) hydrodynamic sizes and PDI of HSA after reaction with different concentrations of KMnO_4_ for 2 h; (e) hydrodynamic sizes of HSA, HSA-MnO_2_ and HSA-MnO_2_-pep; (f) AFM image of HSA-MnO_2_-pep; (g) the changes of hydrodynamic size and PDI of HSA-MnO_2_-pep in PBS over seven days; (h) absorption spectra of HSA-MnO_2_-pep before and after reaction with GSH; (i) the concentration of Mn^2+^ in GSH-treated HSA-MnO_2_-pep solution that was detected by ICP-OES. (For interpretation of the references to colour in this figure legend, the reader is referred to the Web version of this article.)

We then prepared the peptide nanovaccine based on HSA and MnO_2_ for tumor prevention and treatment (see Fig. 1). In order to realize the covalent conjugation between tumor antigenic peptide and HSA, thiol group contained cysteine was added at the N-terminal of the antigenic peptide, which can be covalently linked to the amine group on HSA through SMCC. The successful preparation of HSA-pep can be verified by SDS-PAGE and Coomassie blue staining that the band position of HSA-pep was significantly higher than that of HSA (Fig. 2a). It is well known that DTNB contained Ellman's reagent can be used for the quantification of free thiol groups in peptides and proteins, which is based on the reaction of DTNB with free thiol to form yellow 5′-thio-2-nitrobenzoic acid (TNB) with maximum absorbance at 412 nm. As shown in Fig. 2b, the peptide solution and the solution just mixed with HSA-SMCC showed significant absorption at 412 nm after reacting with DTNB. However, the absorbance of the peptide solution at 412 nm was significantly reduced after first reacting with HSA-SMCC for 4 h and then reacting with DTNB. These demonstrated that the free thiol groups in the solution were significantly reduced and the thiol group contained peptides have been successfully connected to HSA-SMCC, confirming the successful synthesis of HSA-pep. Meanwhile, the calibration curve of thiol group contained peptide was obtained to determine the conjugation efficiency (Fig. S2). After calculation, about 25 peptides were conjugated to each HSA.

Next, HSA-MnO_2_ was prepared through the redox reaction between HSA and KMnO_4_, which was further used for the preparation of HSA-MnO_2_-pep. To optimize the synthesis of HSA-MnO_2_, different concentrations of KMnO_4_ were reacted with HSA at 37 °C for 2 h to prepare HSA-MnO_2_. After reaction, the absorption spectra of the solution were measured. As shown in Fig. 2c, it can be found that the solution showed significant absorption at around 400 nm, which was consistent with the reported literature [32,33]. Taking into account the results of hydrodynamic sizes and absorption spectra, the final concentration of KMnO_4_ was selected as 6 mM (Fig. 2d). After the preparation of HSA-MnO_2_, HSA-MnO_2_-pep was further prepared by covalently conjugating the tumor antigenic peptides to HSA-MnO_2_ through SMCC. The successful preparation of HSA-MnO_2_-pep was characterized by the increase of hydrodynamic size (Fig. 2e) as well as the decrease of free thiol groups measured by Ellman's reagent. It can be calculated by Ellman's reagent that about 25 peptides were conjugated to each HSA-MnO_2_. The morphology of HSA-MnO_2_-pep was imaged by AFM (Fig. 2f). Meanwhile, two peaks at 642.0 eV and 654.0 eV in XPS could be respectively ascribed to Mn_2p3/2_ and Mn_2p1/2_ of MnO_2,_ which can further confirm the successful preparation of HSA-MnO_2_-pep (Fig. S3). Meanwhile, there were no significant changes in hydrodynamic size and PDI of HSA-MnO_2_-pep over seven days (Fig. 2g), indicating its good stability.

Glutathione (GSH) is an important intracellular thiol that participates in many physiological processes. It has been reported that MnO_2_ can be degraded into Mn^2+^ by intracellular GSH. We then verified this process at the solution level. As shown in Fig. 2h and Fig. S4, the absorbance of HSA-MnO_2_-pep solution at around 400 nm was significantly decreased after reaction with GSH, indicating the degradation of MnO_2_ contained in HSA-MnO_2_-pep. Meanwhile, the concentration of Mn^2+^ in HSA-MnO_2_-pep solution measured by inductively coupled plasma atomic emission spectrometer (ICP-OES) was significantly increased after reaction with GSH (Fig. 2i). These phenomena demonstrated that MnO_2_ contained in HSA-MnO_2_-pep can indeed be degraded into Mn^2+^ in the presence of GSH, providing the possibility of acting as immunologic adjuvant to enhance the efficacy of peptide vaccines.

### 3.3 Uptake of nanovaccine by DCs and promoting their maturation

**Fig. 3:**
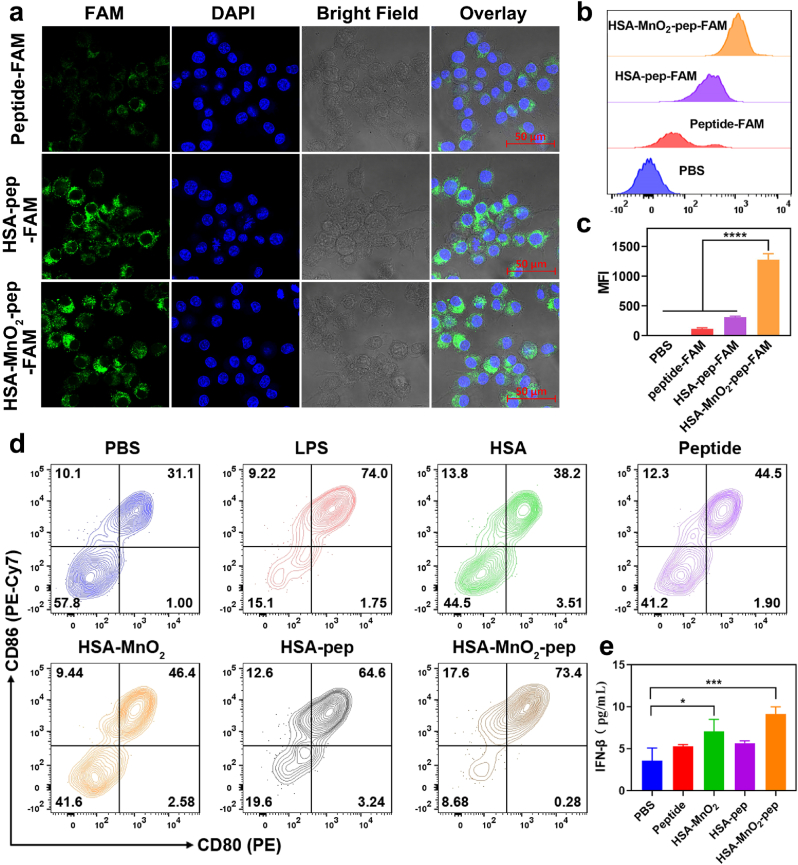
(a) Confocal fluorescent imaging of DC2.4 cells after incubating with peptide-FAM, HSA-pep-FAM or HSA-MnO_2_-pep-FAM (Scale bar: 50 μm); (b) flow cytometry analysis of DC2.4 cells after incubating with peptide-FAM, HSA-pep-FAM or HSA-MnO_2_-pep-FAM; (c) the quantification of corresponding fluorescence signals measured by flow cytometry; (d) flow cytometry analysis of the maturation of BMDCs after incubating with PBS, LPS, HSA, peptide, HSA-MnO_2_, HSA-pep and HSA-MnO_2_-pep for 48 h, respectively; (e) ELISA analysis of IFN-β from BMDCs after incubating with PBS, peptide, HSA-MnO_2_, HSA-pep and HSA-MnO_2_-pep for 24 h, respectively.

Antigen presentation by antigen presenting cells (APCs) is essential for anti-tumor T cell immune responses, and the uptake of nanovaccine by APCs is the prerequisite, which can be investigated by confocal fluorescence imaging. For imaging purpose, HSA-pep-FAM and HSA-MnO_2_-pep-FAM were firstly prepared, and the successful preparation of them could be verified by fluorescent spectra and fluorescence imaging under 488 nm laser (Fig. S5). C57BL/6-derived dendritic cells (DC2.4 cells) were then incubated with peptide-FAM, HSA-pep-FAM and HSA-MnO_2_-pep-FAM respectively. After stained with DAPI, the fluorescence images of the cells were recorded by a confocal fluorescence microscope. As shown in Fig. 3a, it can be clearly seen that the cells after incubated with peptide-FAM showed the weakest fluorescence. While the fluorescence signals of the cells after incubated with HSA-pep-FAM or HSA-MnO_2_-pep-FAM were significantly increased, indicating that the conjugation of tumor antigenic peptides to HSA can promote the uptake of them by APCs. We further used flow cytometry to analyze the uptake of nanovaccine by DC2.4 cells. As shown in Fig. 3b and c, the fluorescence signal in HSA-MnO_2_-pep-FAM treated cells was about 4 times higher than that in HSA-pep-FAM treated cells, which was more than 10 times higher than that in PBS treated cells. This result can further indicate that the conjugation between peptides and HSA can promote the uptake of peptides by APCs. When HSA has been used as the template to prepare HSA-MnO_2_, the further constructed HSA-MnO_2_-pep can be more easily internalized, thus delivering tumor antigenic peptides to APCs more efficiently. The uptake of HSA-MnO_2_-pep-FAM by BMDCs was also investigated by confocal fluorescence imaging and flow cytometry analysis. As shown in Fig. S6, similar results can be observed that HSA-MnO_2_-pep can promote the uptake of antigenic peptides by BMDCs.

The efficient uptake of nanovaccine by DCs might effectively promote DC maturation and antigen presentation. We further investigated the efficacy of promoting BMDCs maturation by measuring the expression levels of CD80 and CD86, which are co-stimulatory molecules expressed on the surface of activated APCs for T cell activation. As shown in Fig. 3d and Fig. S7, Lipopolysaccharide (LPS) can significantly promote the maturation of BMDCs, indicating that BMDCs obtained from mouse bone marrow were normal. Compared with the groups treated with free HSA and peptides, HSA-pep and HSA-MnO_2_-pep can significantly promote the maturation of BMDCs. This may be because HSA as a carrier of antigenic peptides can enhance their immunogenicity. It has been reported that Mn^2+^ degraded from MnO_2_ can promote the activation of STING pathway and further induce DC maturation. To investigate the activation of the STING pathway, the expression levels of its downstream markers phosphorylated TBK1 (p-TBK1) and IFN-β were determined by western blot and ELISA, respectively. Their concentrations in HSA-MnO_2_ treated BMDCs were obviously increased, confirming the activation of STING pathway (Fig. S8 and Fig. 3e). Thus, HSA-MnO_2_ can activate BMDCs to a certain extent. This can also explain why the efficacy of promoting BMDCs maturation by HSA-MnO_2_-pep was better than that by HSA-pep. In other words, the peptide nanovaccine based on HSA and MnO_2_ can effectively activate APCs, which was expected to further trigger the T cell immune responses.

### 3.4 Lymph node accumulation and immune stimulation effects of nanovaccine

**Fig. 4:**
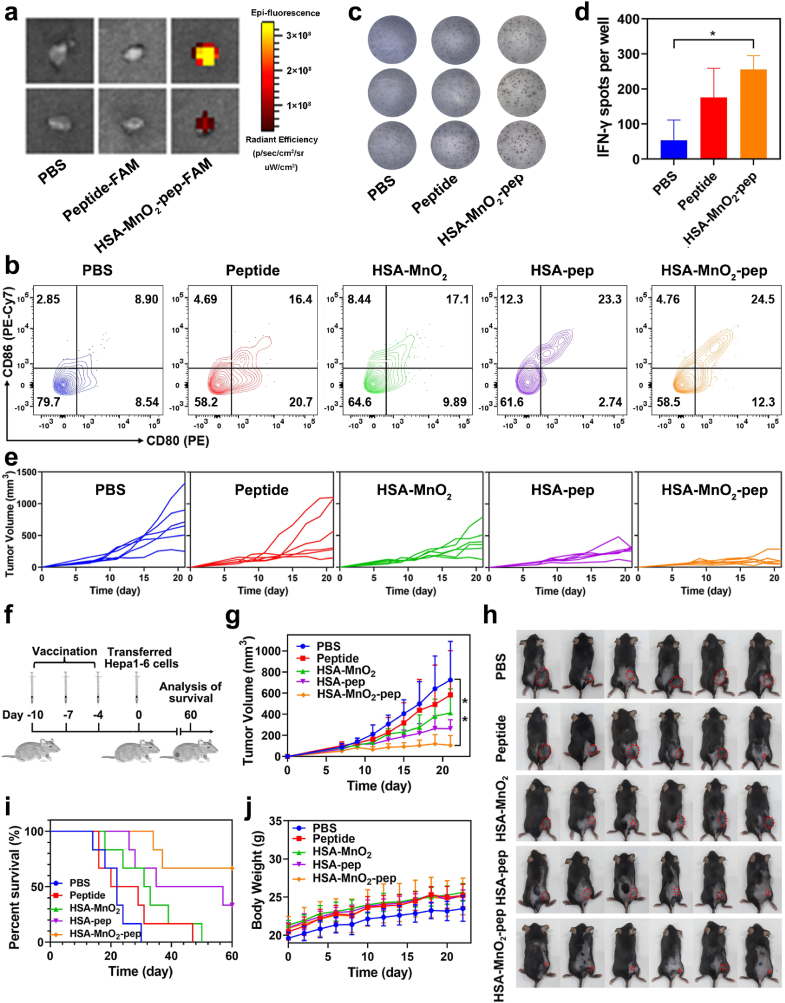
(a) Fluorescence imaging of lymph nodes isolated from the mice after injection with PBS, peptide-FAM or HSA-MnO_2_-pep-FAM for 18 h; (b–c) ELISPOT analysis of IFN-γ production by splenic monocytes from C57BL/6 mice after immunization with PBS, peptide and HSA-MnO_2_-pep, respectively; (c) the quantification of IFN-γ spots; (d) flow cytometry analysis of DCs maturation in lymph nodes three days after last inoculation; (e) tumor volume changes of the mice after different inoculations; (f) flow chart of tumor prevention in mice; (g) changes of tumor volumes after different inoculations; (h) photographs of all the mice when the tumor volume for one of the mice reached 1200 mm^3^; (i) survival statistics of mice after different inoculations; (j) changes of body weight of the mice after different inoculations.

We next investigated the immune stimulation effects of the constructed nanovaccine in mice. The effective accumulation of nanovaccine in lymph nodes was crucial for promoting the maturation of APCs to elicit anti-tumor T-cell immunity. After subcutaneously injecting PBS, peptide-FAM or HSA-MnO_2_-pep-FAM into mice for 18 h, the lymph nodes of these mice were extracted for ex vivo fluorescence imaging. As shown in Fig. 4a, the mice treated with HSA-MnO_2_-pep-FAM showed strong fluorescence in their lymph nodes, while neither the PBS group nor the peptide group showed obvious fluorescence. These indicated that the prepared nanovaccine could effectively accumulate in lymph nodes, which was also the key step for initiating effective anti-tumor immune responses in the body.

The maturation of DCs in lymph nodes was further analyzed by flow cytometry. As shown in Fig. 4b, the proportion of CD80^+^ CD86^+^ DCs in HSA-MnO_2_-pep group was obviously higher than that in peptides and HSA-MnO_2_ treated groups. Meanwhile, the antigen-specific reactivity of splenic T cells measured by ELISPOT assay showed that higher amount of IFN-γ might be secreted in the mice treated with HSA-MnO_2_-pep when compared with the mice treated with free peptides (Fig. 4c and d), indicating the efficient activation of T cells. These findings demonstrated that the constructed nanovaccine can effectively stimulate the maturation of DCs in lymph nodes and trigger T-cell immune responses, which would be further used in tumor prevention and therapy.

### 3.5 The tumor prevention effect of nanovaccine

Encouraged by the above immune stimulation effects, we further evaluated the tumor prevention effect of the prepared nanovaccine, and the flow chart of tumor prevention in C57BL/6 mice was shown in Fig. 4f. After different immunizations, Hepa1-6 cells were subcutaneously injected into the back of each mouse, and their tumor volumes were measured every two days (Fig. 4e and g). It can be found that the mice immunized with free peptides or HSA-MnO_2_ still suffered from obvious tumor growth. Perhaps it is because HSA improved the stability and immunogenicity of the antigenic peptide, the mice immunized with HSA-pep can suppress the tumor growth to a certain degree. Specifically, the mice immunized with HSA-MnO_2_-pep significantly inhibited tumor growth, indicating that the combination of HSA-pep and MnO_2_ can further enhance the tumor prevention effect. The photographs of all the mice shown in Fig. 4h can more intuitively demonstrate that the prepared HSA-MnO_2_-pep exerted the most effective tumor prevention. Compared with PBS group, the survival time of the mice inoculated with HSA-MnO_2_-pep was also significantly prolonged (Fig. 4i). Meanwhile, there was no significant decrease in body weight of the mice in each group, indicating that the constructed nanovaccine did not have obvious toxic or side effects (Fig. 4j).

### 3.6 The therapeutic effect of nanovaccine on tumors

**Fig. 5:**
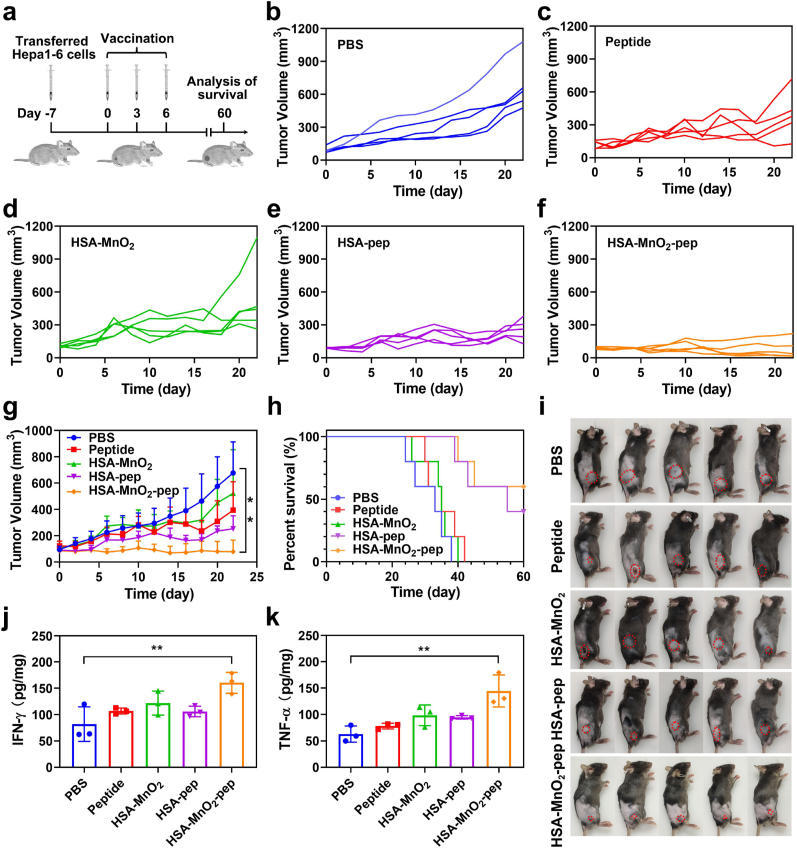
(a) Flow chart of tumor treatment in mice; (b–f) tumor volume changes of the mice after different treatments; (g) changes of average tumor volume in different experimental groups; (h) survival statistics of the mice after different treatments; (i) photographs of all the mice when the tumor volume for one of the mice reached 1200 mm^3^; ELISA analysis of (j) IFN-γ and (k) TNF-α in the tumors from the mice after indicated treatments.

Based on the efficient tumor prevention of HSA-MnO_2_-pep, we further investigated its therapeutic effect on tumors, and the flow chart was shown in Fig. 5a. The tumor-bearing mice were firstly obtained by subcutaneously injecting with Hepa1-6 cells and then subjected to different treatments. The body weight and tumor volume of each mouse were measured every two days. It can be seen from Fig. 5b–g that the mice treated with free peptides or HSA-MnO_2_ can delay tumor growth to a certain extent. While the mice treated with HSA-pep can more obviously suppress tumor growth. As expected, the mice treated with HSA-MnO_2_-pep show the most effective antitumor effect due to the combination of HSA-pep and MnO_2_. The photographs of the mice shown in Fig. 5i can more intuitively demonstrate the anti-tumor effect of HSA-MnO_2_-pep. Furthermore, the survival rate of the mice treated with HSA-MnO_2_-pep was significantly improved (Fig. 5h), with no obvious toxic or side effects that there was no significant difference in body weight changes between the mice treated with PBS and treated with HSA-MnO_2_-pep (Fig. S9).

**Fig. 6:**
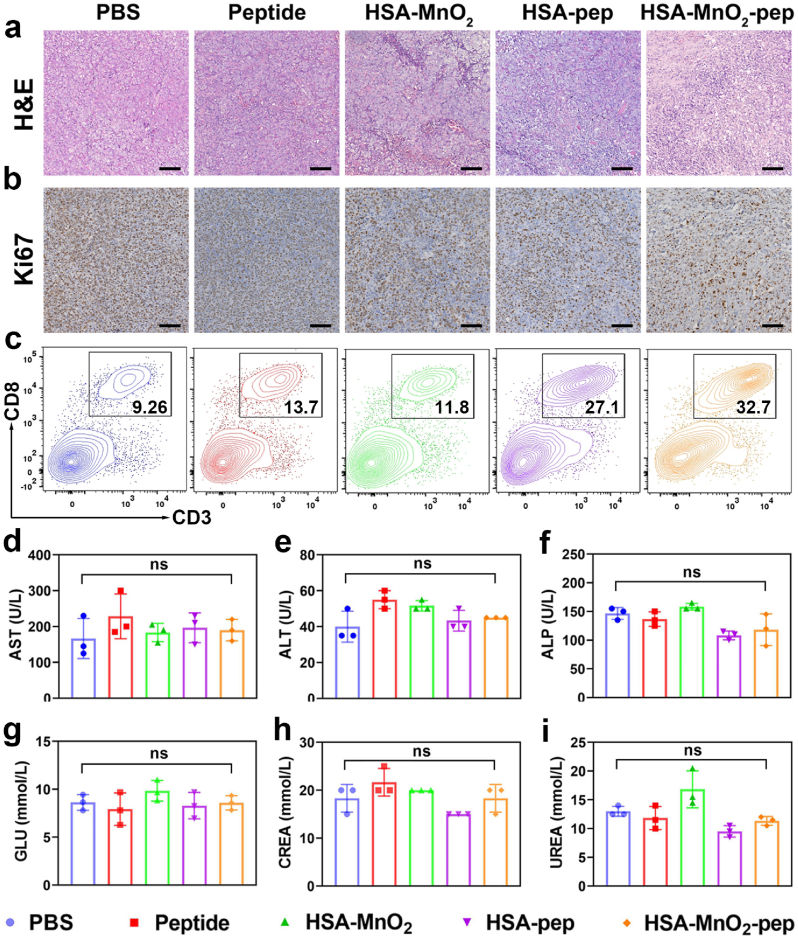
(a) H&E and (b) Ki67 staining of tumor tissue sections from the mice after indicated treatments (Scale bar: 100 μm); (c) flow cytometry analysis of CD8^+^ T cells in the tumors from different treated mice; the analysis of (d) AST, (e) ALT, (f) ALP, (g) GLU, (h) CREA and (i) UREA in serum from the mice after indicated treatments (n = 3).

The antitumor efficiency of HSA-MnO_2_-pep was further confirmed by immunohistochemical staining of tumor slices after different treatments. For H&E staining, the tumor cells of the mice treated with HSA-MnO_2_-pep showed the most significant damage including cell shrinkage and contact loss (Fig. 6a). The tumor slices obtained from different groups also underwent Ki67 staining, since Ki67 can be used as the marker of cell proliferation. As shown in Fig. 6b, the expression of Ki67 in HSA-MnO_2_-pep treated group was obviously decreased, which indicated that the proliferation of tumor cells in this group was decreased. All these results demonstrated that the prepared HSA-MnO_2_-pep can be used for antitumor immunotherapy.

In order to confirm that the anti-tumor effect of HSA-MnO_2_-pep was due to triggered T cell immune response, we further investigated the intratumoral infiltration of T cells after different treatments. On the third day after last injection, the single cell suspensions from the tumors of different groups were obtained for flow cytometry analysis (Fig. 6c and Fig. S10). As expected, the proportion of CD8^+^ T cells in tumors after treated with HSA-pep was significantly higher than that in tumors after treated with PBS. When the tumor-bearing mice were treated with HSA-MnO_2_-pep, the proportion of CD8^+^ T cells in tumors was further increased, which may be due to the effective activation of T cells by the combination of HSA-pep and MnO_2_. In addition, the levels of proinflammation cytokines in the tumors after different treatments were also measured by ELISA. As shown in Fig. 5j and k, the higher levels of IFN-γ and TNF-α in the tumors after treated with HSA-MnO_2_-pep can further indicate the potent antitumor immune responses. In general, the prepared HSA-MnO_2_-pep can be used as tumor vaccine to elicit robust antitumor T-cell immunity, thereby exerting the effects of tumor prevention and treatment.

### 3.7 Biosafety evaluation of nanovaccine

Good biosafety is a prerequisite of tumor vaccines for clinical application. Thus, we finally assessed the biosafety of the prepared nanovaccine by H&E staining and serum biochemical analysis. On the third day after last injection, the heart, liver, spleen, lung and kidney of the mice from each group were obtained for H&E staining. After different treatments, there was no significant difference in the cell morphologies of the major organs, indicating that the prepared nanovaccine did not cause damage to them (Fig. S11). Sera from each group were also collected for the analysis of serum biochemical indicators to evaluate the biosafety of HSA-MnO_2_-pep. The results shown in Fig. 6d–i indicated that there were no significant fluctuations in the levels of biochemical indicators including AST, ALT, ALP, GLU, CREA and UREA. All these results indicated that the prepared nanovaccine possessed good biosafety, which showed great potential for practical applications.

## 4 Conclusions

In general, in order to overcome the poor stability and weak immunogenicity of tumor antigenic peptides, the STING-activating peptide nanovaccine based on HSA and biodegradable MnO_2_ was constructed for tumor prevention and treatment. In this way, the tumor antigenic peptides can be delivered into APCs more efficiently. Meanwhile, MnO_2_ contained in the nanovaccine can be degraded into Mn^2+^, which can induce DCs to produce type I IFNs and further promote DCs maturation, thereby eliciting potent antitumor T-cell immune responses. The prepared peptide nanovaccine can effectively prevent and inhibit tumor growth, thus prolonging the survival time of the mice. In addition, this nanovaccine had no significant toxic side effects and its preparation was relatively simple, which provided a new idea for the development of peptide vaccines for tumor treatment.

## CRediT authorship contribution statement

**Aixian Zheng:** Formal analysis, Funding acquisition, Investigation, Methodology, Writing – original draft. **Zhaoyu Ning:** Formal analysis, Investigation, Methodology. **Xiaorong Wang:** Formal analysis, Investigation, Methodology. **Zhenli Li:** Methodology, Software. **Yupeng Sun:** Formal analysis, Funding acquisition, Investigation. **Ming Wu:** Investigation, Methodology. **Da Zhang:** Investigation, Methodology. **Xiaolong Liu:** Funding acquisition, Project administration, Supervision, Writing – review & editing. **Jianwu Chen:** Conceptualization, Project administration, Supervision. **Yongyi Zeng:** Funding acquisition, Project administration, Supervision.

## Declaration of competing interest

☑ The authors declare that they have no known competing financial interests or personal relationships that could have appeared to influence the work reported in this paper.

## Data Availability

Data will be made available on request.
