# Supplementary material for: Human serum albumin as the carrier to fabricate STING-activating peptide nanovaccine for antitumor immunotherapy
Source: Mater Today Bio. 2024 Jan 14;25:100955. doi: 10.1016/j.mtbio.2024.100955 (PMC10835291; doi:10.1016/j.mtbio.2024.100955)
Supplement: Multimedia component 1 [file mmc1.doc]

Supplementary Material

**Human Serum Albumin as the Carrier to Fabricate STING-activating Peptide Nanovaccine for Antitumor Immunotherapy**

Aixian Zheng,^a#^ Zhaoyu Ning,^ab#^ Xiaorong Wang,^ab^ Zhenli Li,^a^ Yupeng Sun,^a^ Ming Wu,^a^ Da Zhang,^a^ Xiaolong Liu,^a^* Jianwu Chen,^c^* and Yongyi Zeng^a^*

^a^. The United Innovation of Mengchao Hepatobiliary Technology Key Laboratory of Fujian Province, Mengchao Hepatobiliary Hospital of Fujian Medical University, Fuzhou 350025, P. R. China.

^b^. College of Biological Science and Engineering, Fuzhou University, Fuzhou 350116, P. R. China.

^c.^Department of Radiotherapy, Fujian Medical University Union Hospital, Fuzhou 350004, P. R. China.

# These authors contributed equally to this work.

* Corresponding authors. E-mail: xiaoloong.liu@gmail.com; chenjianwucn@aliyun.com; lamp197311@126.com


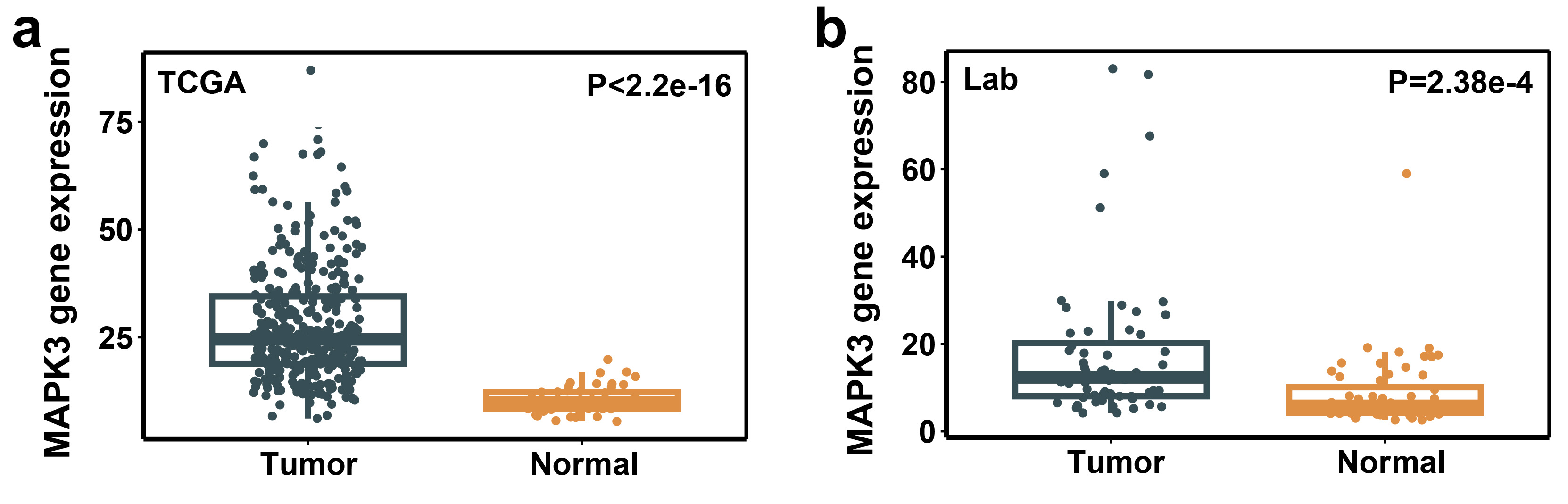


**Figure S1.** (a) The expression levels of MAPK3 gene in liver cancer tissues and normal liver tissues that were analyzed by TCGA database; (b) the expression levels of MAPK3 gene in HCC and paired adjacent tissues that were analyzed by transcriptome sequencing.

**Table S1** Immunogenicity evaluation of MAPK3 derived peptides by NetMHC-4.0

| **Position in MAPK3** | **MHC I** | **Peptide** | **Affinity (nM)** |
| --- | --- | --- | --- |
| 277-285 | H2-Kb | KARNYLQSL | 97.33 |
| 144-152 | H2-Kb | ICYFLYQIL | 110.73 |
| 69-77 | H2-Kb | VAIKKISPF | 171.83 |
| 155-163 | H2-Kb | LKYIHSANV | 230.58 |
| 274-282 | H2-Kb | INMKARNYL | 319.82 |


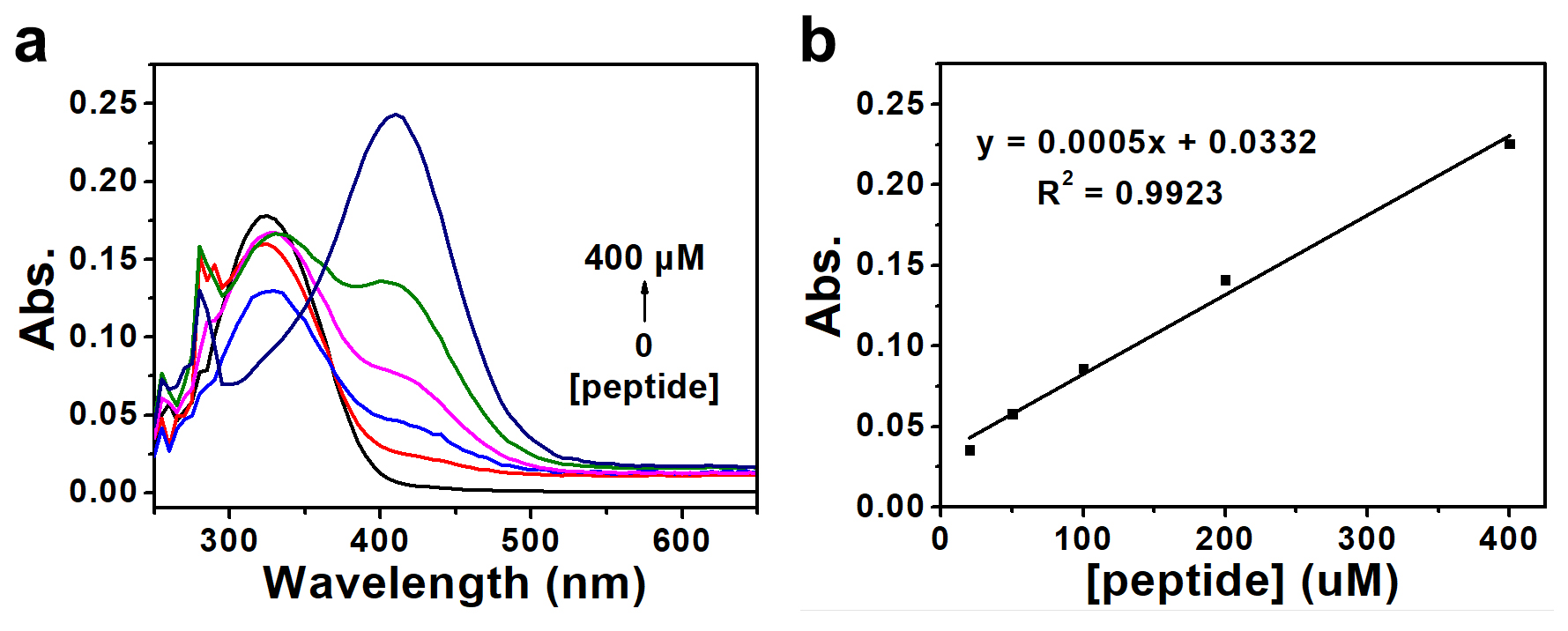


**Figure S2.** (a) Absorption spectra of different concentrations of peptides after reaction with DTNB for 15 min; (b) a linear function for the absorbance intensity of the solution at 412 nm and the concentrations of peptides.


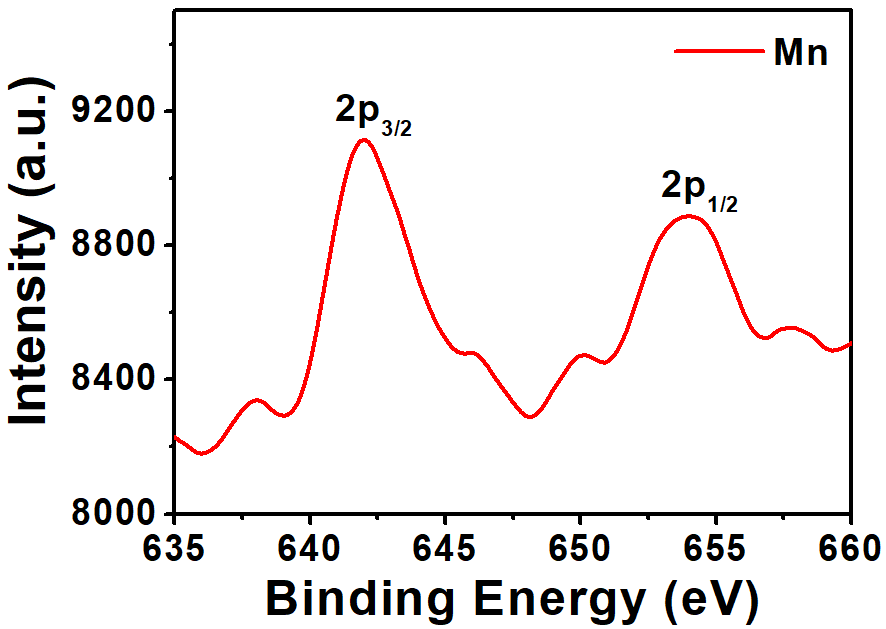


**Figure S3.** The X-ray photoelectron spectroscopy (XPS) of HSA-MnO_2_-pep.


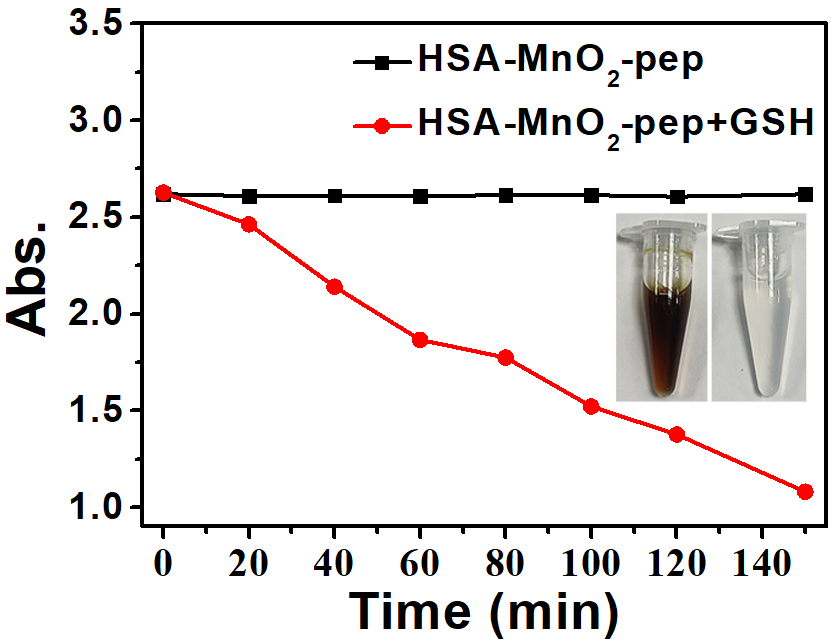


**Figure S4.** Absorbance intensity changes of HSA-MnO_2_-pep and the solution after reaction with GSH over time. The illustration shows HSA-MnO_2_-pep and the solution after reaction with GSH for 150 min.


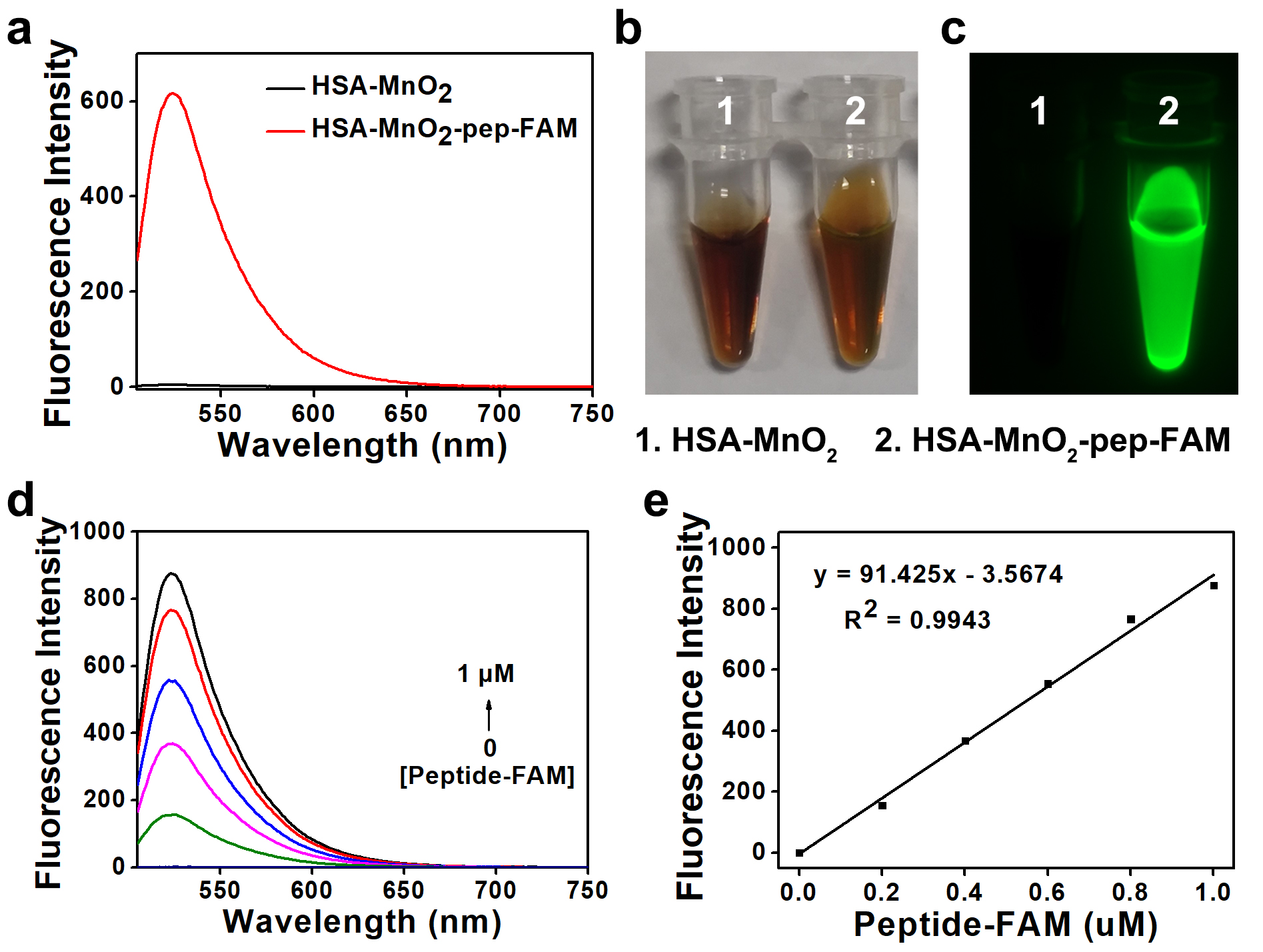


**Figure S5. (**a) Fluorescent emission spectra of HSA-MnO_2_ before and after linking with FAM-labeled peptides; (b) photographs of HSA-MnO_2_ and HSA-MnO_2_-pep-FAM solution; (c) fluorescence imaging of HSA-MnO_2_ and HSA-MnO_2_-pep-FAM solution; (d) fluorescent emission spectra of FAM-labeled peptides at different concentrations; (e) a linear function for the concentrations of FAM-labeled peptides and corresponding fluorescence intensity.


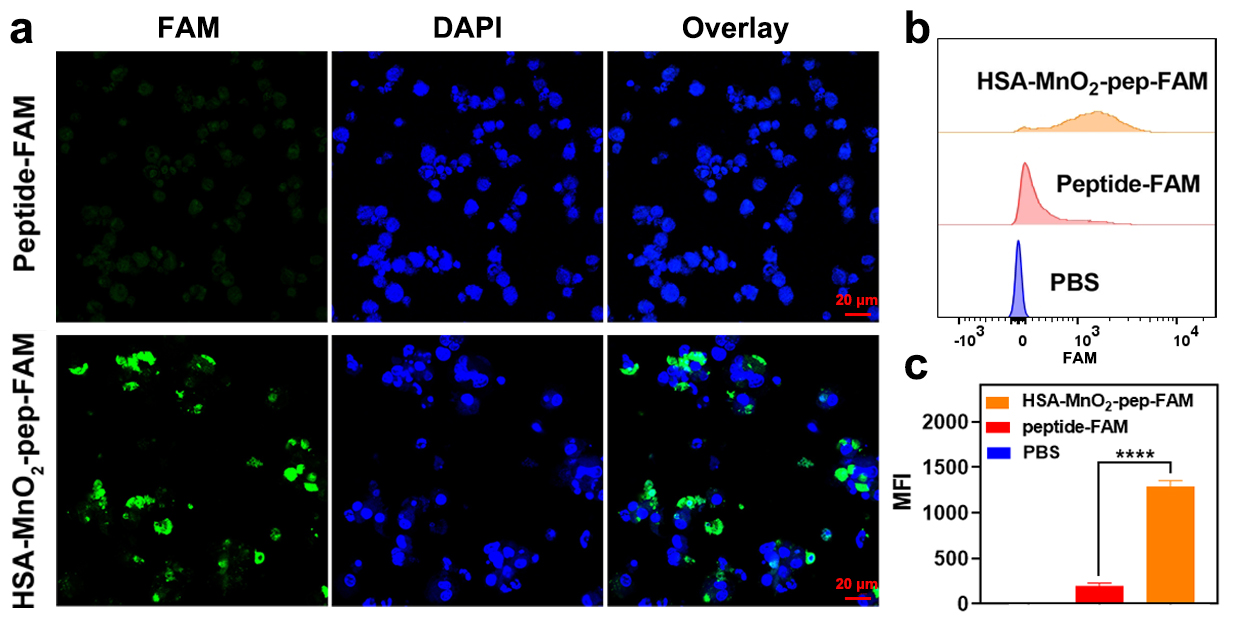


**Figure S6.** (a) Confocal fluorescent imaging of BMDCs after incubating with peptide-FAM or HSA-MnO_2_-pep-FAM (Scale bar: 20 μm); (b) flow cytometry analysis of BMDCs after incubating with peptide-FAM or HSA-MnO_2_-pep-FAM; (c) the quantification of corresponding fluorescence signals measured by flow cytometry.


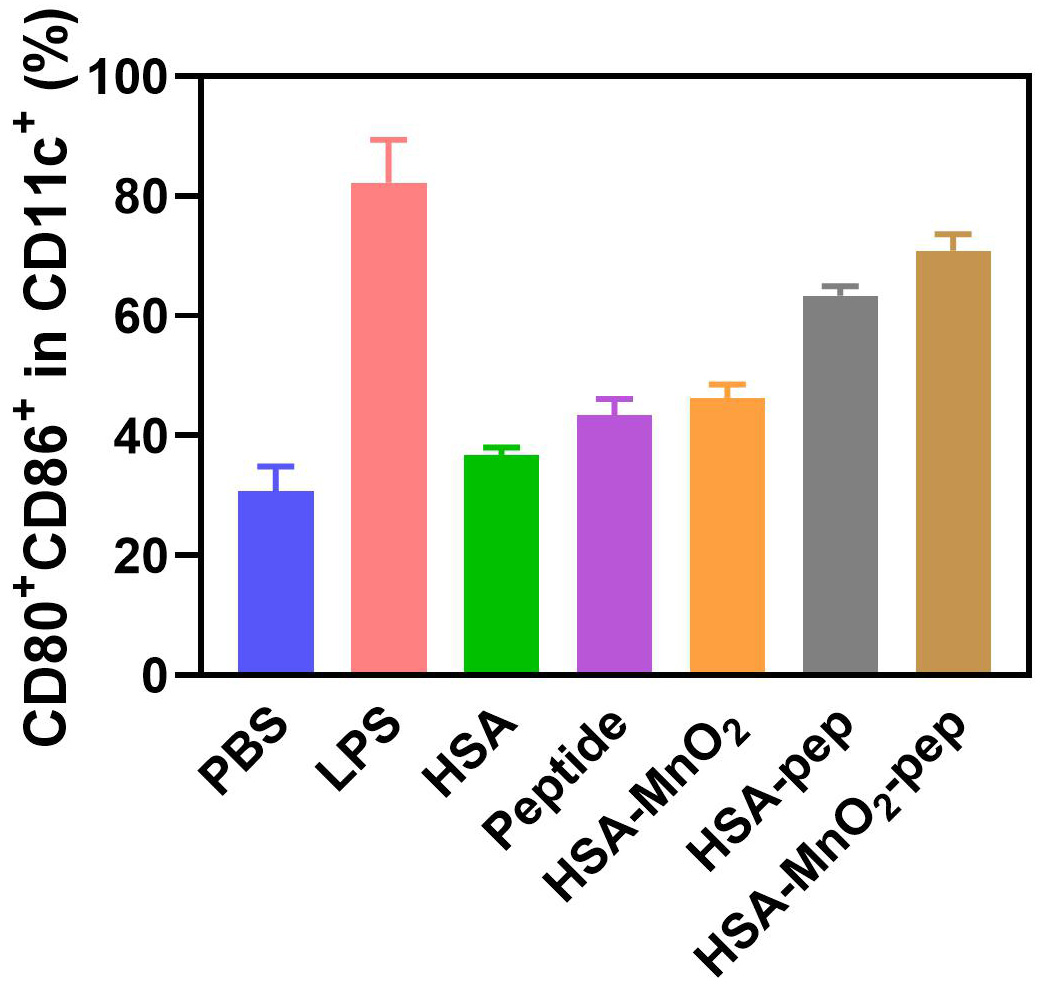


**Figure S7.** Flow cytometry analysis of the maturation of BMDCs after incubating with PBS, LPS, HSA, peptide, HSA-MnO_2_, HSA-pep and HSA-MnO_2_-pep for 48 h, respectively.

*
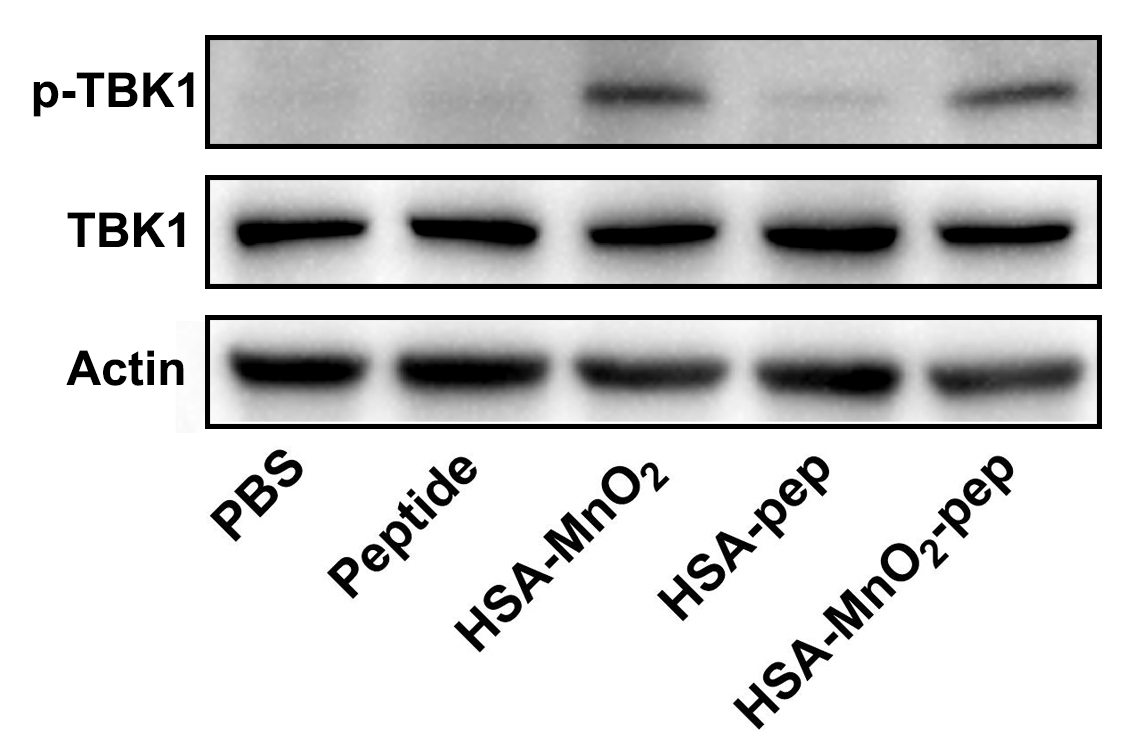
*

**Figure S8.** Western blot analysis of TBK1 and p-TBK1 expressions in BMDCs after incubated with PBS, peptide, HSA-MnO_2_, HSA-pep and HSA-MnO_2_-pep, respectively.


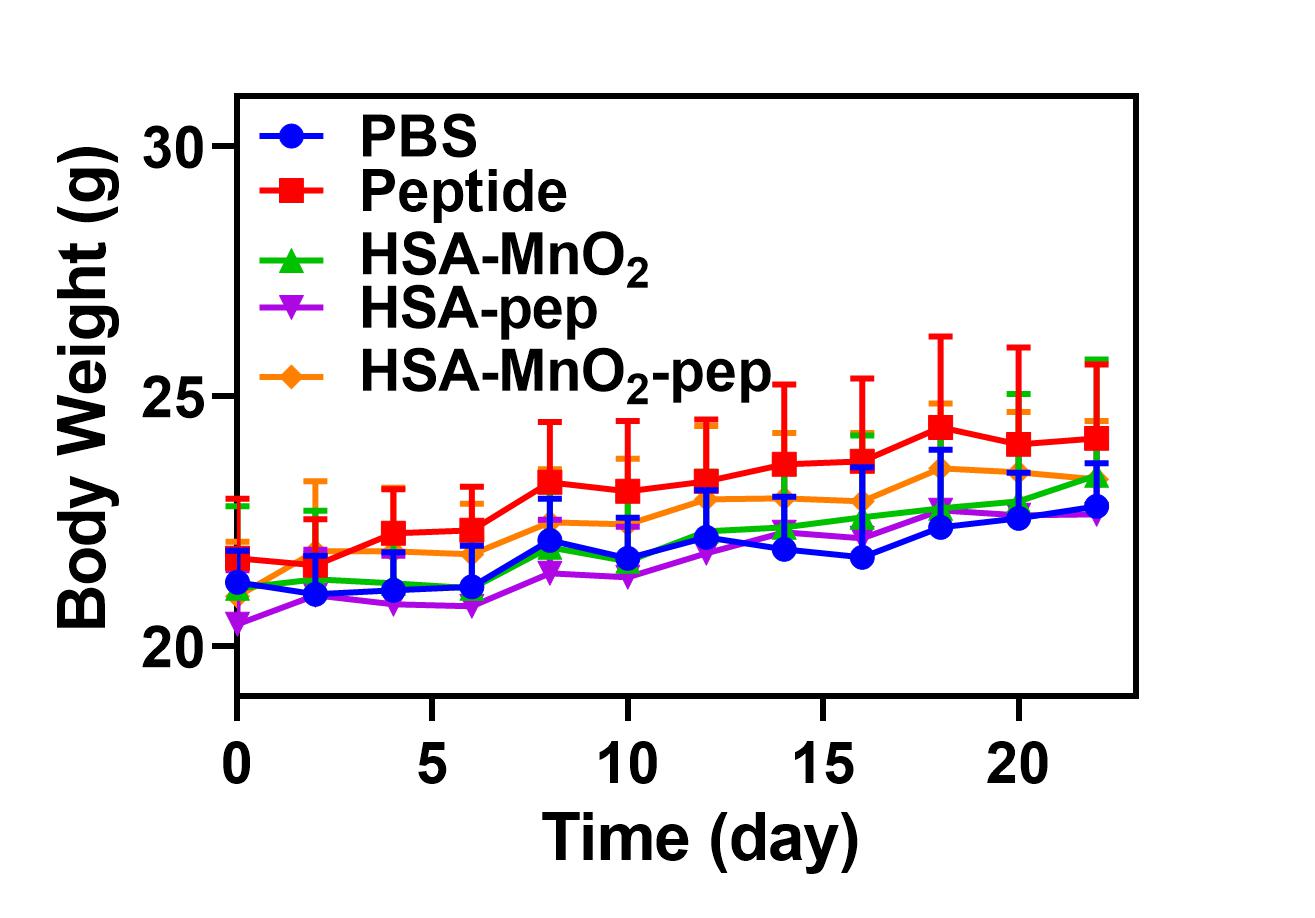


**Figure S9.** Body weight changes of the mice in different experimental groups.


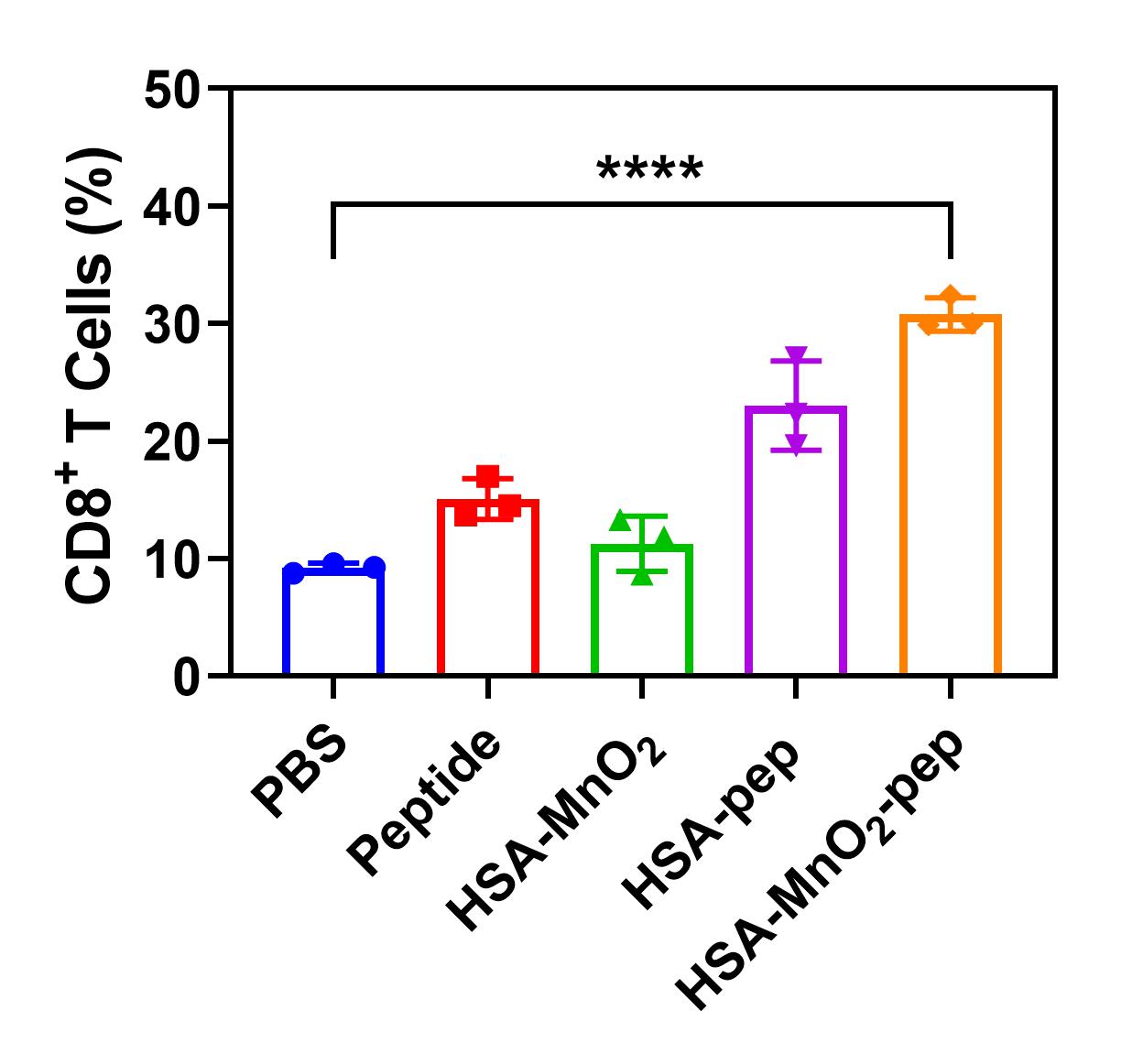


**Figure S10.** Flow cytometry analysis of CD8+ T cells in tumors from different treated mice.


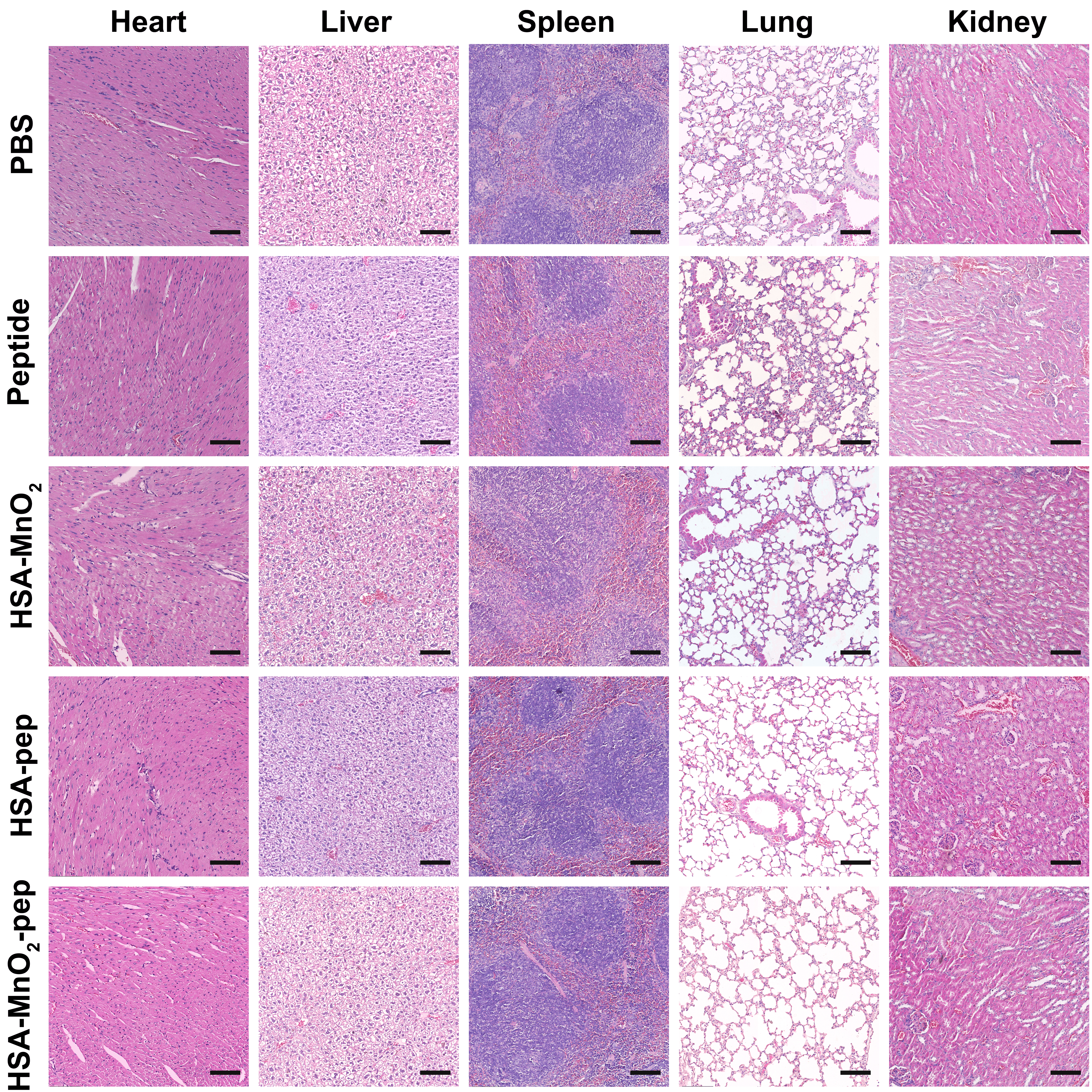


**Figure S11.** H&E staining of major organs (heart, liver, spleen, lung and kidney) from the mice after different treatments (Scale bar: 100 μm).
